# Editorial: Histone modifications in cancer

**DOI:** 10.3389/fphar.2025.1727417

**Published:** 2025-11-17

**Authors:** Jiaqian Luo, Sizhi P. Gao

**Affiliations:** 1 BCMB Program, Weill Cornell Medicine, New York, NY, United States; 2 Human Oncology and Pathogenesis Program, Sloan Kettering Institute, Memorial Sloan Kettering Cancer Center, New York, NY, United States

**Keywords:** histone modification, cancer biology, epigenetics, epigenetic therapies, tumor immune microenvironment, precision oncology, methylation, superenhancer

## 1 Introduction

Cancer remains one of the most challenging diseases in modern medicine due to its intricate molecular mechanisms. Beyond genetic alterations, epigenetic regulation has emerged as a central determinant of tumor initiation, progression, and therapeutic response. Among epigenetic mechanisms, histone modifications, including methylation, acetylation, lactylation, phosphorylation, and ubiquitination, play pivotal roles in modulating chromatin architecture and gene expression program without altering the underlying DNA sequence. These histone marks are dynamically written, erased, and read by specific histone-modifying enzymes and reader proteins that collectively fine-tune transcriptional activities. Among the best characterized are acetylation, regulated by histone acetyltransferases (HATs) and histone deacetylases (HDACs), and methylation, controlled by histone methyltransferases (HMTs) and demethylases (HDMs). These reversible modifications regulate essential oncogenic pathways, shaping tumor progression, immune escape, and responsiveness to therapy.

This Research Topic, Histone Modifications in Cancer, was curated to provide timely insights into the multifaceted roles of histone modifications in cancer biology and their translational potential as therapeutic targets. The Research Topic integrates original research and review articles that advance our understanding of histone-mediated regulatory mechanisms and highlight emerging opportunities for epigenetic intervention in cancer therapy.

## 2 Histone modifications driving cancer progression and therapeutic resistance

Histone-modifying enzymes have been increasingly recognized as critical contributors to tumorigenesis and drug resistance through both epigenetic and non-epigenetic mechanisms (Rodrigues et al., 2024; Stief et al., 2020; Wang et al., 2025). In alignment with this concept, the contribution by Wang et al. demonstrates that chromobox protein homolog 2 (CBX2) promotes glioma progression and chemoresistance. Their study shows that elevated CBX2 expression significantly enhances glioma cell proliferation and resistance to chemotherapy drug temozolomide. Mechanistic analyses reveal that CBX2 recruited enhancer of zeste homolog 2, (*EZH2*) to induce H3K27me3-mediated epigenetic silencing of the phosphatase and tensin homolog (*PTEN)* promoter. This study underscores the oncogenic function of histone methylation and highlights how a dysregulated epigenetic landscape can undermine therapeutic efficacy in glioma.

In parallel, Wu et al. investigated the clinically relevant traditional Chinese medicine norcantharidin and elucidated a histone methylationdependent mechanism underlying its antitumor activity. This work reports that epigenetic silencing of topoisomerase IIα (*TOP2A*) gene directly shapes oncogenic transcriptional programs in hepatocellular carcinoma. The Polycomb Repressive Complex 2 (PRC2)-induced deposition of the repressive histone mark H3K27me3 at the *TOP2A* promoter significantly disrupts the cell proliferation and induced G2/M phase cell cycle arrest. Taken together, these fundamental studies expand our understanding of histone modifications in driving tumor progression and therapeutic resistance, while identifying promising new therapeutic targets such as CBX2 and the PRC2 complex to counteract epigenetic dysfunction and enhance the efficacy of anti-tumor therapies.

## 3 Histone modifications shaping tumor-immune interface

Immunotherapy has emerged as a revolutionary treatment option for many cancers that were previously considered untreatable or poorly responsive to conventional modalities such as surgery, radiotherapy, and chemotherapy. In recent years, increasing attention has been directed toward the role of histone modifications in modulating the tumor-immune microenvironment. Both the activity of immune cells involved in antitumor responses and the immunogenicity of tumor cells can be profoundly influenced by histone modification-mediated regulation. Moreover, accumulating evidence indicates that epigenetic drugs can modulate immune pathways to prevent immune evasion and enhance tumor immunogenicity (Liu et al., 2022; Zhang et al., 2025). These insights provide strong rationale for exploring the combination of epigenetic-targeted therapies with established immunotherapy drugs. In support of this, Spadotto et al. report that HDAC6 inhibition by ITF3756 modulates PD-L1 expression and alters monocyte phenotype, thereby enhancing the efficacy of immune checkpoint blockade in colon cancer preclinical models. These findings highlight the intersection of histone acetylation and immune regulation, nominating ITF3756 as a selective HDAC6 inhibitor and immunomodulator. Moreover, this study reinforces the therapeutic promise of epigenetic-immunotherapy combinations to overcome adaptive resistance and minimize toxicity.

## 4 Emerging translational opportunities in histone modifications

Therapeutic targeting of histone-modifying enzymes has become an area of intense investigation, with several agents already approved for clinical use and many others currently advancing through various phases of clinical trials. Histone deacetylase (HDAC) inhibitors such as vorinostat and romidepsin have been approved for cutaneous T-cell lymphoma (El Omari et al., 2025). In addition, the EZH2 inhibitor tazemetostat has been approved for epithelioid sarcoma and follicular lymphoma and a growing number of EZH2 inhibitors and degraders are progressing through clinical trials across various cancer types (Tang et al., 2025). These advances demonstrate the clinical feasibility of targeting histone modifications and highlight opportunities to expand such strategies into a broader range of malignancies.

In addition to significant mechanistic investigations in this Research Topic, the review from Li et al. provides a comprehensive summary of super-enhancers in liver cancer, providing insights from pathogenic mechanisms to clinical applications. This work emphasizes how enhancer-associated histone modifications govern transcriptional dependencies in tumor cells, while also underscoring their translational value as biomarkers and therapeutic vulnerabilities. This offers the translational perspectives on histone-associated regulatory elements, providing insights for establishing epigenetic biomarkers based on histone modifications to advance precision oncology.

## 5 Conclusion and outlook

By exploring histone modifications in cancer from mechanistic, immunologic, and translational therapeutic perspectives, this Research Topic reflects the breadth of epigenetic regulation in tumor biology. In particular, these studies demonstrate how histone marks can shape responses to immune checkpoint blockade, reveal chromatin-associated elements such as super-enhancers as critical regulatory factors, and establish their translational value as both biomarkers and therapeutic targets. By bridging fundamental epigenetic mechanisms with emerging clinical opportunities, this body of work provides a framework for understanding histone modifications within broader regulatory networks that define malignant phenotypes and therapeutic vulnerabilities.

As research on histone modifications continues to expand, several directions hold promise for advancing both mechanistic understanding and clinical translation. Identifying predictive histone modification signatures capable of guiding precision oncology strategies remains an urgent priority. The integration of multi-omics data, encompassing histone modification landscapes, chromatin accessibility, genomic, transcriptomic, and proteomic profiles will be essential for refining biomarker discovery and predicting therapeutic outcomes. Parallel efforts to develop highly selective and potent agents targeting histone modification-related enzymes and regulatory proteins should be accompanied by rigorous preclinical and clinical evaluation of efficacy and toxicity. Furthermore, combination approaches that pair epigenetic therapies with current therapy regimens, such as chemotherapy, targeted agents, immunotherapies, or antibody-drug conjugates, offer promising opportunities to overcome resistance and extend durable benefit to patients. We hope this Research Topic will inspire continued investigations into the epigenetic dimensions of cancer biology and help establish histone modification research as a critical frontier in precision oncology and a driver of innovative antitumor drug discovery.
